# Supplementary material for: Effect of the Temperature on the Process of Preferential Solvation of 1,4-Dioxane, 12-Crown-4, 15-Crown-5 and 18-Crown-6 Ethers in the Mixture of N-Methylformamide with Water: Composition of the Solvation Shell of the Cyclic Ethers
Source: Int J Mol Sci. 2023 May 18;24(10):8934. doi: 10.3390/ijms24108934 (PMC10218947; doi:10.3390/ijms24108934)
Supplement: Supplementary file 1 [file ijms-24-08934-s001.zip › ijms-2379817-supplementary.pdf]

Effect of the Temperature on the Process of Preferential Solvation of 1,4-Dioxane, 12-Crown-4, 15-Crown-5 and 18-Crown-6 Ethers in the Mixture of *N*-Methylformamide with Water: Composition of the Solvation Shell of the Cyclic Ethers

Małgorzata Jóźwiak*, Monika A. Trzmielak, Michał Wasiak

University of Lodz, Faculty of Chemistry, Department of Physical Chemistry, Pomorska 165, 90-236 Lodz, Poland

*corresponding author: malgorzata.jozwiak@chemia.uni.lodz.pl

Supplementary Materials

Table S1. Total number of water (W) and *N*-methylformamide (NMF) molecules in the solvation sphere of 1,4-dioxane r = (r_W + r_{NMF}), mole fraction of *N*-methylformamide (NMF) in the solvation sphere of the solute (y_{NMF}), entropic factor of the transfer entropy of 1,4-dioxane in the process of preferential solvation at T = 293.15 K in dependency of the mole fraction of water (x_W) or *N*-methylformamide (x_{NMF}) in the mixture W + NMF.

1,4-dioxane								
water (W) + *N*-methylformamide (NMF) T = 293.15 K								
x_W	r	r_W	r_{NMF}	x_{NMF}	y_{NMF}	$T\Delta_{tr}S$ / kJ·mol^{-1} $(W \rightarrow NMF + W)_{(B)}$	$T\Delta_{tr}S$ / kJ·mol^{-1} $(W \rightarrow NMF + W)_{(A)}$	δ^a
0.000	1.4	0.000	1.000	1.000	1.000	0.000	0.000	0.000
0.100	1.4	0.015	0.985	0.900	0.985	−0.206	−0.204	0.002
0.200	1.4	0.034	0.976	0.800	0.966	−0.419	−0.417	0.002
0.300	1.4	0.057	0.943	0.700	0.944	−0.640	−0.638	0.002
0.400	1.4	0.085	0.915	0.600	0.914	−0.864	−0.866	−0.002
0.500	1.4	0.123	0.877	0.500	0.877	−1.094	−1.094	0.000
0.600	1.4	0.174	0.826	0.400	0.826	−1.312	−1.312	0.000
0.700	1.4	0.246	0.754	0.300	0.754	−1.490	−1.492	−0.002
0.800	1.4	0.359	0.641	0.200	0.641	−1.564	−1.566	−0.002
0.900	1.4	0.558	0.442	0.100	0.442	−1.332	−1.334	−0.002
0.920	1.4	0.617	0.383	0.080	0.383	−1.205	−1.206	−0.001
0.940	1.4	0.687	0.313	0.060	0.313	−1.028	−1.030	−0.002
0.960	1.4	0.771	0.229	0.040	0.229	−0.789	−0.789	0.000
0.980	1.4	0.873	0.127	0.020	0.127	−0.458	−0.458	0.000
1.000	1.4	1.000	0.000	0.000	0.000	0.000	0.000	0.000

$^a\delta = T\Delta_{tr}S(W \rightarrow NMF + W)_{(B)} - T\Delta_{tr}S(W \rightarrow NMF + W)_{(A)}$

Table S2. Total number of water (W) and *N*-methylformamide (NMF) molecules in the solvation sphere of 1,4-dioxane $r = (r_W + r_{NMF})$, mole fraction of *N*-methylformamide (NMF) in the solvation sphere of the solute (y_{NMF}), entropic factor of the transfer entropy of 1,4-dioxane in the process of preferential solvation at T = 298.15 K in dependency of the mole fraction of water (x_W) or *N*-methylformamide (x_{NMF}) in the mixture W + NMF.

1,4-dioxane								
water (W) + *N*-methylformamide (NMF) T = 298.15 K								
x_W	r	r_W	r_{NMF}	x_{NMF}	y_{NMF}	$T\Delta_{tr}S$ / kJ·mol^{-1} $(W \rightarrow NMF + W)_{(B)}$	$T\Delta_{tr}S$ / kJ·mol^{-1} $(W \rightarrow NMF + W)_{(A)}$	δ^a
0.00	1.3	0.000	1.300	1.000	1.000	0.000	0.000	0.000
0.10	1.3	0.019	1.281	0.900	0.985	−0.197	−0.200	−0.003
0.20	1.3	0.041	1.259	0.800	0.968	−0.409	−0.409	0.000
0.30	1.3	0.069	1.231	0.700	0.947	−0.626	−0.628	−0.002
0.40	1.3	0.104	1.196	0.600	0.920	−0.852	−0.854	−0.002
0.50	1.3	0.150	1.150	0.500	0.885	−1.081	−1.083	−0.002
0.60	1.3	0.212	1.088	0.400	0.837	−1.306	−1.306	0.000
0.70	1.3	0.303	0.997	0.300	0.767	−1.494	−1.496	−0.002
0.80	1.3	0.445	0.855	0.200	0.658	−1.587	−1.588	−0.001
0.90	1.3	0.701	0.599	0.100	0.461	−1.378	−1.379	−0.001
0.92	1.3	0.779	0.521	0.080	0.401	−1.253	−1.253	0.000
0.94	1.3	0.872	0.428	0.060	0.329	−1.077	−1.077	−0.000
0.96	1.3	0.984	0.316	0.040	0.243	−0.834	−0.831	0.003
0.98	1.3	1.124	0.176	0.020	0.135	−0.485	−0.487	−0.002
1.00	1.3	1.300	0.000	0.000	0.000	0.000	0.000	0.000

$^a\delta = T\Delta_{tr}S(W \rightarrow NMF + W)_{(B)} - T\Delta_{tr}S(W \rightarrow NMF + W)_{(A)}$

Table S3. Total number of water (W) and *N*-methylformamide (NMF) molecules in the solvation sphere of 1,4-dioxane $r = (r_W + r_{NMF})$, mole fraction of *N*-methylformamide (NMF) in the solvation sphere of the solute (y_{NMF}), entropic factor of the transfer entropy of 1,4-dioxane in the process of preferential solvation at T = 303.15 K in dependency of the mole fraction of water (x_W) or *N*-methylformamide (x_{NMF}) in the mixture W + NMF.

1,4-dioxane								
water (W) + *N*-methylformamide (NMF) T = 303.15 K								
x_W	r	r_W	r_{NMF}	x_{NMF}	y_{NMF}	$T\Delta_{tr}S$ / kJ·mol^{-1} $(W \rightarrow NMF + W)_{(B)}$	$T\Delta_{tr}S$ / kJ·mol^{-1} $(W \rightarrow NMF + W)_{(A)}$	δ^a
0.00	1.2	0.000	1.200	1.000	1.000	0.000	0.000	0.000
0.10	1.2	0.016	1.184	0.900	0.987	−0.193	−0.194	−0.001
0.20	1.2	0.035	1.165	0.800	0.971	−0.398	−0.399	−0.001
0.30	1.2	0.059	1.141	0.700	0.951	−0.612	−0.613	−0.001
0.40	1.2	0.089	1.111	0.600	0.926	−0.837	−0.837	0.000
0.50	1.2	0.129	1.071	0.500	0.893	−1.064	−1.067	−0.003
0.60	1.2	0.183	1.017	0.400	0.848	−1.293	−1.292	0.001
0.70	1.2	0.263	0.937	0.300	0.781	−1.489	−1.492	−0.004
0.80	1.2	0.389	0.811	0.200	0.676	−1.603	−1.602	0.001
0.90	1.2	0.623	0.577	0.100	0.481	−1.420	−1.419	0.001
0.92	1.2	0.696	0.504	0.080	0.420	−1.297	−1.298	−0.001
0.94	1.2	0.783	0.417	0.060	0.348	−1.126	−1.124	0.002
0.96	1.2	0.891	0.309	0.040	0.258	−0.873	−0.875	−0.002
0.98	1.2	1.026	0.174	0.020	0.145	−0.516	−0.518	−0.002
1.00	1.2	1.200	0.000	0.000	0.000	0.000	0.000	0.000

$^a\delta = T\Delta_{tr}S(W \rightarrow NMF + W)_{(B)} - T\Delta_{tr}S(W \rightarrow NMF + W)_{(A)}$

Table S4. Total number of water (W) and *N*-methylformamide (NMF) molecules in the solvation sphere of 1,4-dioxane $r = (r_W + r_{NMF})$, mole fraction of *N*-methylformamide (NMF) in the solvation sphere of the solute (y_{NMF}), entropic factor of the transfer entropy of 1,4-dioxane in the process of preferential solvation at T = 308.15 K in dependency of the mole fraction of water (x_W) or *N*-methylformamide (x_{NMF}) in the mixture W + NMF.

1,4-dioxane								
water (W) + *N*-methylformamide (NMF) T = 308.15 K								
x_W	r	r_W	r_{NMF}	x_{NMF}	y_{NMF}	$T\Delta_{tr}S$ / kJ·mol^{-1} $(W \rightarrow NMF + W)_{(B)}$	$T\Delta_{tr}S$ / kJ·mol^{-1} $(W \rightarrow NMF + W)_{(A)}$	δ^a
0.00	1.2	0.000	1.200	1.000	1.000	0.000	0.000	0.000
0.10	1.2	0.016	1.184	0.900	0.987	−0.196	−0.197	−0.001
0.20	1.2	0.035	1.165	0.800	0.971	−0.405	−0.405	0.000
0.30	1.2	0.059	1.141	0.700	0.951	−0.622	−0.624	−0.002
0.40	1.2	0.089	1.111	0.600	0.926	−0.850	−0.851	−0.001
0.50	1.2	0.129	1.071	0.500	0.893	−1.082	−1.084	−0.002
0.60	1.2	0.183	1.017	0.400	0.848	−1.314	−1.314	0.000
0.70	1.2	0.263	0.937	0.300	0.781	−1.514	−1.517	−0.003
0.80	1.2	0.389	0.811	0.200	0.676	−1.630	−1.629	0.001
0.90	1.2	0.623	0.577	0.100	0.481	−1.443	−1.443	0.000
0.92	1.2	0.696	0.504	0.080	0.420	−1.319	−1.320	−0.001
0.94	1.2	0.783	0.417	0.060	0.348	−1.144	−1.142	0.002
0.96	1.2	0.891	0.309	0.040	0.258	−0.888	−0.889	−0.001
0.98	1.2	1.026	0.174	0.020	0.145	−0.524	−0.527	−0.003
1.00	1.2	1.200	0.000	0.000	0.000	0.000	0.000	0.000

$^a\delta = T\Delta_{tr}S(W \rightarrow NMF + W)_{(B)} - T\Delta_{tr}S(W \rightarrow NMF + W)_{(A)}$

Table S5. Total number of water (W) and *N*-methylformamide (NMF) molecules in the solvation sphere of 12C4 $r = (r_W + r_{NMF})$, mole fraction of *N*-methylformamide (NMF) in the solvation sphere of the solute (y_{NMF}), entropic factor of the transfer entropy of 12C4 in the process of preferential solvation at $T = 293.15$ K in dependency of the mole fraction of water (x_W) or *N*-methylformamide (x_{NMF}) in the mixture W + NMF.

12C4								
water (W) + *N*-methylformamide (NMF) $T = 293.15$ K								
x_W	r	r_W	r_{NMF}	x_{NMF}	y_{NMF}	$T\Delta_{tr}S$ / kJ·mol^{-1} $(W \rightarrow NMF + W)_{(B)}$	$T\Delta_{tr}S$ / kJ·mol^{-1} $(W \rightarrow NMF + W)_{(A)}$	δ^a
0.00	11.5	0.000	11.500	1.000	1.000	0.000	0.000	0.000
0.10	11.5	0.442	11.058	0.900	0.962	−0.753	−0.752	0.001
0.20	11.5	0.950	10.550	0.800	0.917	−1.473	−1.475	−0.002
0.30	11.5	1.537	9.963	0.700	0.866	−2.148	−2.148	0.000
0.40	11.5	2.226	9.274	0.600	0.806	−2.746	−2.746	0.000
0.50	11.5	3.044	8.456	0.500	0.735	−3.230	−3.230	0.000
0.60	11.5	4.032	7.468	0.400	0.649	−3.540	−3.539	0.001
0.70	11.5	5.250	6.250	0.300	0.543	−3.582	−3.582	0.000
0.80	11.5	6.787	4.713	0.200	0.410	−3.209	−3.209	0.000
0.90	11.5	8.788	2.712	0.100	0.236	−2.167	−2.167	0.000
0.92	11.5	9.263	2.237	0.080	0.195	−1.843	−1.844	−0.001
0.94	11.5	9.768	1.732	0.060	0.151	−1.472	−1.472	0.000
0.96	11.5	10.307	1.193	0.040	0.104	−1.045	−1.045	0.000
0.98	11.5	10.883	0.617	0.020	0.054	−0.557	−0.557	0.000
1.00	11.5	11.500	0.000	0.000	0.000	0.000	0.000	0.000

[a] $\delta = T\Delta_{tr}S(W \rightarrow NMF + W)_{(B)} - T\Delta_{tr}S(W \rightarrow NMF + W)_{(A)}$

Table S6. Total number of water (W) and *N*-methylformamide (NMF) molecules in the solvation sphere of 12C4 r = (r_W + r_{NMF}), mole fraction of *N*-methylformamide (NMF) in the solvation sphere of the solute (y_{NMF}), entropic factor of the transfer entropy of 12C4 in the process of preferential solvation at T = 298.15 K in dependency of the mole fraction of water (x_W) or *N*-methylformamide (x_{NMF}) in the mixture W + NMF.

12C4								
water (W) + *N*-methylformamide (NMF) T = 298.15 K								
x_W	r	r_W	r_{NMF}	x_{NMF}	y_{NMF}	$T\Delta_{tr}S$ / kJ·mol^{-1} $(W \rightarrow NMF + W)_{(B)}$	$T\Delta_{tr}S$ / kJ·mol^{-1} $(W \rightarrow NMF + W)_{(A)}$	δ^a
0.00	9.9	0.000	9.900	1.000	1.000	0.000	0.000	0.000
0.10	9.9	0.360	9.540	0.900	0.964	−0.713	−0.712	0.001
0.20	9.9	0.776	9.124	0.800	0.922	−1.399	−1.400	−0.001
0.30	9.9	1.259	8.641	0.700	0.873	−2.048	−2.048	0.000
0.40	9.9	1.829	8.071	0.600	0.815	−2.631	−2.630	0.001
0.50	9.9	2.512	7.388	0.500	0.746	−3.110	−3.110	0.000
0.60	9.9	3.344	6.556	0.400	0.662	−3.430	−3.431	−0.001
0.70	9.9	4.380	5.520	0.300	0.558	−3.500	−3.501	−0.001
0.80	9.9	5.705	4.195	0.200	0.424	−3.168	−3.168	0.000
0.90	9.9	7.462	2.438	0.100	0.246	−2.166	−2.167	−0.001
0.92	9.9	7.884	2.016	0.080	0.204	−1.849	−1.849	0.000
0.94	9.9	8.335	1.565	0.060	0.158	−1.481	−1.481	0.000
0.96	9.9	8.819	1.081	0.040	0.109	−1.056	−1.055	0.001
0.98	9.9	9.339	0.561	0.020	0.057	−0.565	−0.564	0.001
1.00	9.9	9.900	0.000	0.000	0.000	0.000	0.000	0.000

$^a\delta = T\Delta_{tr}S(W \rightarrow NMF + W)_{(B)} - T\Delta_{tr}S(W \rightarrow NMF + W)_{(A)}$

Table S7. Total number of water (W) and *N*-methylformamide (NMF) molecules in the solvation sphere of 12C4 $r = (r_W + r_{NMF})$, mole fraction of *N*-methylformamide (NMF) in the solvation sphere of the solute (y_{NMF}), entropic factor of the transfer entropy of 12C4 in the process of preferential solvation at T = 303.15 K in dependency of the mole fraction of water (x_W) or *N*-methylformamide (x_{NMF}) in the mixture W + NMF.

12C4								
water (W) + *N*-methylformamide (NMF) T = 303.15 K								
x_W	r	r_W	r_{NMF}	x_{NMF}	y_{NMF}	$T\Delta_{tr}S$ / kJ · mol^{-1} $(W \rightarrow NMF + W)_{(B)}$	$T\Delta_{tr}S$ / kJ · mol^{-1} $(W \rightarrow NMF + W)_{(A)}$	δ^a
0.00	8.5	0.000	8.500	1.000	1.000	0.000	0.000	0.000
0.10	8.5	0.283	8.217	0.900	0.967	−0.696	−0.696	0.000
0.20	8.5	0.611	7.889	0.800	0.928	−1.378	−1.377	0.001
0.30	8.5	0.997	7.503	0.700	0.883	−2.026	−2.026	0.000
0.40	8.5	1.456	7.044	0.600	0.829	−2.621	−2.622	−0.001
0.50	8.5	2.011	6.489	0.500	0.763	−3.128	−3.127	0.001
0.60	8.5	2.698	5.802	0.400	0.683	−3.485	−3.485	0.000
0.70	8.5	3.568	4.932	0.300	0.580	−3.601	−3.602	−0.001
0.80	8.5	4.705	3.795	0.200	0.446	−3.314	−3.313	0.001
0.90	8.5	6.257	2.243	0.100	0.264	−2.316	−2.315	0.001
0.92	8.5	6.638	1.862	0.080	0.219	−1.986	−1.986	0.000
0.94	8.5	7.049	1.451	0.060	0.171	−1.598	−1.599	−0.001
0.96	8.5	7.493	1.007	0.040	0.118	−1.145	−1.146	−0.001
0.98	8.5	7.975	0.525	0.020	0.062	−0.617	−0.617	0.000
1.0	8.5	8.500	0.000	0.000	0.000	0.000	0.000	0.000

[a] $\delta = T\Delta_{tr}S(W \rightarrow NMF + W)_{(B)} - T\Delta_{tr}S(W \rightarrow NMF + W)_{(A)}$

Table S8. Total number of water (W) and *N*-methylformamide (NMF) molecules in the solvation sphere of 12C4 $r = (r_W + r_{NMF})$, mole fraction of *N*-methylformamide (NMF) in the solvation sphere of the solute (y_{NMF}), entropic factor of the transfer entropy of 12C4 in the process of preferential solvation at $T = 308.15$ K in dependency of the mole fraction of water (x_W) or *N*-methylformamide (x_{NMF}) in the mixture W + NMF.

12C4								
water (W) + *N*-methylformamide (NMF) $T = 308.15$ K								
x_W	r	r_W	r_{NMF}	x_{NMF}	y_{NMF}	$T\Delta_{tr}S$ / kJ·mol^{-1} $(W \rightarrow NMF + W)_{(B)}$	$T\Delta_{tr}S$ / kJ·mol^{-1} $(W \rightarrow NMF + W)_{(A)}$	δ^a
0.00	7.2	0.000	7.200	1.000	1.000	0.000	0.000	0.000
0.10	7.2	0.225	6.975	0.900	0.969	−0.645	−0.646	−0.001
0.20	7.2	0.487	6.713	0.800	0.932	−1.281	−1.281	0.000
0.30	7.2	0.796	6.404	0.700	0.889	−1.894	−1.894	0.000
0.40	7.2	1.166	6.034	0.600	0.838	−2.465	−2.463	0.002
0.50	7.2	1.619	5.581	0.500	0.775	−2.954	−2.955	−0.001
0.60	7.2	2.183	5.017	0.400	0.697	−3.317	−3.318	−0.001
0.70	7.2	2.906	4.294	0.300	0.596	−3.459	−3.460	−0.001
0.80	7.2	3.867	3.333	0.200	0.463	−3.219	−3.220	−0.001
0.90	7.2	5.206	1.994	0.100	0.277	−2.284	−2.286	−0.002
0.92	7.2	5.539	1.661	0.080	0.231	−1.968	−1.968	0.000
0.94	7.2	5.901	1.299	0.060	0.180	−1.591	−1.591	0.000
0.96	7.2	6.295	0.905	0.040	0.126	−1.147	−1.145	0.002
0.98	7.2	6.727	0.473	0.020	0.066	−0.618	−0.619	−0.001
1.00	7.2	7.200	0.000	0.000	0.000	0.000	0.000	0.000

[a] $\delta = T\Delta_{tr}S(W \rightarrow NMF + W)_{(B)} - T\Delta_{tr}S(W \rightarrow NMF + W)_{(A)}$

Table S9. Total number of water (W) and *N*-methylformamide (NMF) molecules in the solvation sphere of 15C5 $r = (r_W + r_{NMF})$, mole fraction of *N*-methylformamide (NMF) in the solvation sphere of the solute (y_{NMF}), entropic factor of the transfer entropy of 15C5 in the process of preferential solvation at T = 293.15 K in dependency of the mole fraction of water (x_W) or *N*-methylformamide (x_{NMF}) in the mixture W + NMF.

15C5								
water (W) + *N*-methylformamide (NMF) T = 293.15 K								
x_W	r	r_W	r_{NMF}	x_{NMF}	y_{NMF}	$T\Delta_{tr}S$ / kJ·mol^{-1} $(W \rightarrow NMF + W)_{(B)}$	$T\Delta_{tr}S$ / kJ·mol^{-1} $(W \rightarrow NMF + W)_{(A)}$	δ^a
0.00	20.7	0.000	20.700	1.000	1.000	0.000	0.000	0.000
0.10	20.7	0.923	19.777	0.900	0.955	−1.063	−1.063	0.000
0.20	20.7	1.967	18.733	0.800	0.905	−2.062	−2.062	0.000
0.30	20.7	3.158	17.542	0.700	0.847	−2.967	−2.968	−0.001
0.40	20.7	4.528	16.172	0.600	0.781	−3.744	−3.743	0.001
0.50	20.7	6.123	14.577	0.500	0.704	−4.333	−4.334	−0.001
0.60	20.7	8.001	12.699	0.400	0.613	−4.662	−4.663	−0.001
0.70	20.7	10.245	10.455	0.300	0.505	−4.618	−4.617	0.001
0.80	20.7	12.976	7.724	0.200	0.373	−4.027	−4.027	0.000
0.90	20.7	16.369	4.331	0.100	0.209	−2.631	−2.630	0.001
0.92	20.7	17.149	3.551	0.080	0.172	−2.221	−2.220	0.001
0.94	20.7	17.969	2.731	0.060	0.132	−1.758	−1.758	0.000
0.96	20.7	18.832	1.868	0.040	0.090	−1.237	−1.237	0.000
0.98	20.7	19.741	0.959	0.020	0.046	−0.653	−0.654	−0.001
1.00	20.7	20.700	0.000	0.000	0.000	0.000	0.000	0.000

[a] $\delta = T\Delta_{tr}S(W \rightarrow NMF + W)_{(B)} - T\Delta_{tr}S(W \rightarrow NMF + W)_{(A)}$

Table S10. Total number of water (W) and *N*-methylformamide (NMF) molecules in the solvation sphere of 15C5 $r = (r_W + r_{NMF})$, mole fraction of *N*-methylformamide (NMF) in the solvation sphere of the solute (y_{NMF}), entropic factor of the transfer entropy of 15C5 in the process of preferential solvation at T = 298.15 K in dependency of the mole fraction of water (x_W) or *N*-methylformamide (x_{NMF}) in the mixture W + NMF.

15C5								
water (W) + *N*-methylformamide (NMF) T = 298.15 K								
x_W	r	r_W	r_{NMF}	x_{NMF}	y_{NMF}	$T\Delta_{tr}S$ / kJ·mol^{-1} $(W \rightarrow NMF + W)_{(B)}$	$T\Delta_{tr}S$ / kJ·mol^{-1} $(W \rightarrow NMF + W)_{(A)}$	δ^a
0.00	16.7	0.000	16.700	1.000	1.000	0.000	0.000	0.000
0.10	16.7	0.677	16.023	0.900	0.959	−1.026	−1.027	−0.001
0.20	16.7	1.449	15.251	0.800	0.913	−2.005	−2.005	0.000
0.30	16.7	2.339	14.361	0.700	0.860	−2.909	−2.910	−0.001
0.40	16.7	3.376	13.324	0.600	0.798	−3.702	−3.703	−0.001
0.50	16.7	4.599	12.101	0.500	0.725	−4.330	−4.331	−0.001
0.60	16.7	6.063	10.637	0.400	0.637	−4.716	−4.716	0.000
0.70	16.7	7.848	8.852	0.300	0.530	−4.738	−4.737	0.001
0.80	16.7	10.073	6.627	0.200	0.397	−4.204	−4.204	0.000
0.90	16.7	12.922	3.778	0.100	0.226	−2.805	−2.805	0.000
0.92	16.7	13.590	3.110	0.080	0.186	−2.381	−2.380	0.001
0.94	16.7	14.298	2.402	0.060	0.144	−1.895	−1.894	0.001
0.96	16.7	15.050	1.650	0.040	0.099	−1.340	−1.341	−0.001
0.98	16.7	15.849	0.851	0.020	0.051	−0.712	−0.712	0.000
1.00	16.7	16.700	0.000	0.000	0.000	0.000	0.000	0.000

$^a\delta = T\Delta_{tr}S(W \rightarrow NMF + W)_{(B)} - T\Delta_{tr}S(W \rightarrow NMF + W)_{(A)}$

Table S11. Total number of water (W) and *N*-methylformamide (NMF) molecules in the solvation sphere of 15C5 $r = (r_W + r_{NMF})$, mole fraction of *N*-methylformamide (NMF) in the solvation sphere of the solute (y_{NMF}), entropic factor of the transfer entropy of 15C5 in the process of preferential solvation at T = 303.15 K in dependency of the mole fraction of water (x_W) or *N*-methylformamide (x_{NMF}) in the mixture W + NMF.

15C5								
water (W) + *N*-methylformamide (NMF) T = 303.15 K								
x_W	r	r_W	r_{NMF}	x_{NMF}	y_{NMF}	$T\Delta_{tr}S$ / kJ·mol^{-1} $(W \rightarrow NMF + W)_{(B)}$	$T\Delta_{tr}S$ / kJ·mol^{-1} $(W \rightarrow NMF + W)_{(A)}$	δ^a
0.00	12.9	0.000	12.900	1.000	1.000	0.000	0.000	0.000
0.10	12.9	0.456	12.444	0.900	0.965	−0.981	−0.980	0.001
0.20	12.9	0.983	11.917	0.800	0.924	−1.931	−1.931	0.000
0.30	12.9	1.598	11.302	0.700	0.876	−2.831	−2.829	0.002
0.40	12.9	2.326	10.574	0.600	0.820	−3.644	−3.643	0.001
0.50	12.9	3.201	9.699	0.500	0.752	−4.320	−4.321	−0.001
0.60	12.9	4.271	8.629	0.400	0.669	−4.783	−4.782	0.001
0.70	12.9	5.612	7.288	0.300	0.565	−4.899	−4.900	−0.001
0.80	12.9	7.340	5.560	0.200	0.431	−4.456	−4.457	−0.001
0.90	12.9	9.651	3.249	0.100	0.252	−3.069	−3.070	−0.001
0.92	12.9	10.210	2.690	0.080	0.209	−2.623	−2.624	−0.001
0.94	12.9	10.809	2.091	0.060	0.162	−2.105	−2.105	0.000
0.96	12.9	11.454	1.446	0.040	0.112	−1.502	−1.502	0.000
0.98	12.9	12.149	0.751	0.020	0.058	−0.804	−0.805	−0.001
1.00	12.9	12.900	0.000	0.000	0.000	0.000	0.000	0.000

$^a\delta = T\Delta_{tr}S(W \rightarrow NMF + W)_{(B)} - T\Delta_{tr}S(W \rightarrow NMF + W)_{(A)}$

Table S12. Total number of water (W) and *N*-methylformamide (NMF) molecules in the solvation sphere of 15C5 $r = (r_W + r_{NMF})$, mole fraction of *N*-methylformamide (NMF) in the solvation sphere of the solute (y_{NMF}), entropic factor of the transfer entropy of 15C5 in the process of preferential solvation at T = 308.15 K in dependency of the mole fraction of water (x_W) or *N*-methylformamide (x_{NMF}) in the mixture W + NMF.

15C5								
water (W) + *N*-methylformamide (NMF) T = 308.15 K								
x_W	r	r_W	r_{NMF}	x_{NMF}	y_{NMF}	$T\Delta_{tr}S$ / kJ·mol^{-1} $(W \rightarrow NMF + W)_{(B)}$	$T\Delta_{tr}S$ / kJ·mol^{-1} $(W \rightarrow NMF + W)_{(A)}$	δ^a
0.00	10.4	0.000	10.400	1.000	1.000	0.000	0.000	0.000
0.10	10.4	0.325	10.075	0.900	0.969	−0.932	−0.933	−0.001
0.20	10.4	0.703	9.697	0.800	0.932	−1.851	−1.851	0.000
0.30	10.4	1.150	9.250	0.700	0.889	−2.735	−2.736	−0.001
0.40	10.4	1.685	8.715	0.600	0.838	−3.557	−3.558	−0.001
0.50	10.4	2.338	8.062	0.500	0.775	−4.269	−4.269	0.000
0.60	10.4	3.153	7.247	0.400	0.697	−4.792	−4.793	−0.001
0.70	10.4	4.197	6.203	0.300	0.596	−4.999	−4.998	0.001
0.80	10.4	5.585	4.815	0.200	0.463	−4.651	−4.651	0.000
0.90	10.4	7.519	2.881	0.100	0.277	−3.302	−3.301	0.001
0.92	10.4	8.001	2.399	0.080	0.231	−2.842	−2.843	−0.001
0.94	10.4	8.524	1.876	0.060	0.180	−2.298	−2.298	0.000
0.96	10.4	9.093	1.307	0.040	0.126	−1.656	−1.654	0.002
0.98	10.4	9.716	0.684	0.020	0.066	−0.896	−0.895	0.001
1.00	10.4	10.400	0.000	0.000	0.000	0.000	0.000	0.000

$^a\delta = T\Delta_{tr}S(W \rightarrow NMF + W)_{(B)} - T\Delta_{tr}S(W \rightarrow NMF + W)_{(A)}$

Table S13. Total number of water (W) and *N*-methylformamide (NMF) molecules in the solvation sphere of 18C6 r = (r_W + r_{NMF}), mole fraction of *N*-methylformamide (NMF) in the solvation sphere of the solute (y_{NMF}), entropic factor of the transfer entropy of 18C6 in the process of preferential solvation at T = 293.15 K in dependency of the mole fraction of water (x_W) or *N*-methylformamide (x_{NMF}) in the mixture W + NMF.

18C6								
water (W) + *N*-methylformamide (NMF) T = 293.15 K								
x_W	r	r_W	r_{NMF}	x_{NMF}	y_{NMF}	$T\Delta_{tr}S$ / kJ·mol^{-1} $(W \rightarrow NMF+W)_{(B)}$	$T\Delta_{tr}S$ / kJ·mol^{-1} $(W \rightarrow NMF+W)_{(A)}$	δ^a
0.00	68.3	0.000	68.300	1.000	1.000	0.000	0.000	0.000
0.10	68.3	4.068	64.232	0.900	0.940	−1.743	−1.743	0.000
0.20	68.3	8.519	59.781	0.800	0.875	−3.298	−3.298	0.000
0.30	68.3	13.409	54.891	0.700	0.804	−4.620	−4.620	0.000
0.40	68.3	18.807	49.493	0.600	0.725	−5.653	−5.652	0.001
0.50	68.3	24.797	43.503	0.500	0.637	−6.324	−6.324	0.000
0.60	68.3	31.481	36.819	0.400	0.539	−6.544	−6.545	−0.001
0.70	68.3	38.987	29.313	0.300	0.429	−6.198	−6.199	−0.001
0.80	68.3	47.477	20.823	0.200	0.305	−5.136	−5.136	0.000
0.90	68.3	57.158	11.142	0.100	0.163	−3.158	−3.158	0.000
0.92	68.3	59.260	9.040	0.080	0.132	−2.630	−2.631	−0.001
0.94	68.3	61.422	6.878	0.060	0.101	−2.054	−2.054	0.000
0.96	68.3	63.647	4.653	0.040	0.068	−1.426	−1.425	0.001
0.98	68.3	65.939	2.361	0.020	0.035	−0.742	−0.742	0.000
1.00	68.3	68.300	0.000	0.000	0.000	0.000	0.000	0.000

$^a\delta = T\Delta_{tr}S(W \rightarrow NMF+W)_{(B)} - T\Delta_{tr}S(W \rightarrow NMF+W)_{(A)}$

Table S14. Total number of water (W) and *N*-methylformamide (NMF) molecules in the solvation sphere of 18C6 $r = (r_W + r_{NMF})$, mole fraction of *N*-methylformamide (NMF) in the solvation sphere of the solute (y_{NMF}), entropic factor of the transfer entropy of 18C6 in the process of preferential solvation at T = 298.15 K in dependency of the mole fraction of water (x_W) or *N*-methylformamide (x_{NMF}) in the mixture W + NMF.

18C6								
water (W) + *N*-methylformamide (NMF) T = 298.15 K								
x_W	r	r_W	r_{NMF}	x_{NMF}	y_{NMF}	$T\Delta_{tr}S$ / kJ·mol^{-1} $(W \rightarrow NMF + W)_{(B)}$	$T\Delta_{tr}S$ / kJ·mol^{-1} $(W \rightarrow NMF + W)_{(A)}$	δ^a
0.00	53.8	0.000	53.800	1.000	1.000	0.000	0.000	0.000
0.10	53.8	2.992	50.808	0.900	0.944	−1.711	−1.711	0.000
0.20	53.8	6.294	47.506	0.800	0.883	−3.260	−3.259	0.001
0.30	53.8	9.958	43.842	0.700	0.815	−4.598	−4.597	0.001
0.40	53.8	14.046	39.754	0.600	0.739	−5.668	−5.668	0.000
0.50	53.8	18.637	35.163	0.500	0.654	−6.395	−6.395	0.000
0.60	53.8	23.828	29.972	0.400	0.557	−6.681	−6.681	0.000
0.70	53.8	29.746	24.054	0.300	0.447	−6.396	−6.396	0.001
0.80	53.8	36.556	17.244	0.200	0.321	−5.364	−5.363	0.001
0.90	53.8	44.476	9.324	0.100	0.173	−3.343	−3.343	0.000
0.92	53.8	46.217	7.583	0.080	0.141	−2.793	−2.793	0.000
0.94	53.8	48.017	5.783	0.060	0.107	−2.188	−2.187	0.001
0.96	53.8	49.879	3.921	0.040	0.073	−1.522	−1.523	−0.001
0.98	53.8	51.805	1.995	0.020	0.037	−0.795	−0.795	0.000
1.00	53.8	53.800	0.000	0.000	0.000	0.000	0.000	0.000

$^a\delta = T\Delta_{tr}S(W \rightarrow NMF + W)_{(B)} - T\Delta_{tr}S(W \rightarrow NMF + W)_{(A)}$

Table S15. Total number of water (W) and *N*-methylformamide (NMF) molecules in the solvation sphere of 18C6 $r = (r_W + r_{NMF})$, mole fraction of *N*-methylformamide (NMF) in the solvation sphere of the solute (y_{NMF}), entropic factor of the transfer entropy of 18C6 in the process of preferential solvation at T = 303.15 K in dependency of the mole fraction of water (x_W) or *N*-methylformamide (x_{NMF}) in the mixture W + NMF.

18C6								
water (W) + *N*-methylformamide (NMF) T = 303.15 K								
x_W	r	r_W	r_{NMF}	x_{NMF}	y_{NMF}	$T\Delta_{tr}S$ / kJ·mol^{-1} $(W \rightarrow NMF + W)_{(B)}$	$T\Delta_{tr}S$ / kJ·mol^{-1} $(W \rightarrow NMF + W)_{(A)}$	δ^a
0.00	41.7	0.000	41.700	1.000	1.000	0.000	0.000	0.000
0.10	41.7	2.111	39.589	0.900	0.949	−1.707	−1.707	0.001
0.20	41.7	4.468	37.232	0.800	0.893	−3.276	−3.277	−0.001
0.30	41.7	7.115	34.585	0.700	0.829	−4.663	−4.664	−0.001
0.40	41.7	10.109	31.591	0.600	0.758	−5.808	−5.808	0.000
0.50	41.7	13.524	28.176	0.500	0.676	−6.628	−6.628	0.000
0.60	41.7	17.456	24.244	0.400	0.581	−7.012	−7.013	−0.001
0.70	41.7	22.030	19.670	0.300	0.472	−6.811	−6.810	0.001
0.80	41.7	27.419	14.281	0.200	0.342	−5.807	−5.806	0.001
0.90	41.7	33.862	7.838	0.100	0.188	−3.689	−3.690	−0.001
0.92	41.7	35.304	6.396	0.080	0.153	−3.096	−3.096	0.000
0.94	41.7	36.806	4.894	0.060	0.117	−2.435	−2.435	0.000
0.96	41.7	38.369	3.331	0.040	0.080	−1.704	−1.703	0.001
0.98	41.7	39.999	1.701	0.020	0.041	−0.894	−0.893	0.001
1.00	41.7	41.700	0.000	0.000	0.000	0.000	0.000	0.000

$^a\delta = T\Delta_{tr}S(W \rightarrow NMF + W)_{(B)} - T\Delta_{tr}S(W \rightarrow NMF + W)_{(A)}$

Table S16. Total number of water (W) and *N*-methylformamide (NMF) molecules in the solvation sphere of 18C6 $r = (r_W + r_{NMF})$, mole fraction of *N*-methylformamide (NMF) in the solvation sphere of the solute (y_{NMF}), entropic factor of the transfer entropy of 18C6 in the process of preferential solvation at T = 308.15 K in dependency of the mole fraction of water (x_W) or *N*-methylformamide (x_{NMF}) in the mixture W + NMF.

18C6								
water (W) + *N*-methylformamide (NMF) T = 308.15 K								
x_W	r	r_W	r_{NMF}	x_{NMF}	y_{NMF}	$T\Delta_{tr}S$ / kJ·mol^{-1} $(W \rightarrow NMF + W)_{(B)}$	$T\Delta_{tr}S$ / kJ·mol^{-1} $(W \rightarrow NMF + W)_{(A)}$	δ^a
0.00	33.7	0.000	33.700	1.000	1.000	0.000	0.000	0.000
0.10	33.7	1.571	32.129	0.900	0.953	−1.671	−1.672	−0.001
0.20	33.7	3.340	30.360	0.800	0.901	−3.230	−3.231	−0.001
0.30	33.7	5.347	28.353	0.700	0.841	−4.633	−4.634	−0.001
0.40	33.7	7.643	26.057	0.600	0.773	−5.820	−5.819	0.001
0.50	33.7	10.297	23.403	0.500	0.694	−6.705	−6.704	0.001
0.60	33.7	13.399	20.301	0.400	0.602	−7.171	−7.171	0.000
0.70	33.7	17.072	16.628	0.300	0.493	−7.052	−7.053	−0.001
0.80	33.7	21.490	12.210	0.200	0.362	−6.102	−6.103	−0.001
0.90	33.7	26.906	6.794	0.100	0.202	−3.947	−3.947	0.000
0.92	33.7	28.139	5.561	0.080	0.165	−3.325	−3.325	0.000
0.94	33.7	29.431	4.269	0.060	0.127	−2.626	−2.626	0.000
0.96	33.7	30.785	2.915	0.040	0.086	−1.844	−1.844	0.000
0.98	33.7	32.206	1.494	0.020	0.044	−0.972	−0.972	0.000
1.00	33.7	33.700	0.000	0.000	0.000	0.000	0.000	0.000

[a] $\delta = T\Delta_{tr}S(W \rightarrow NMF + W)_{(B)} - T\Delta_{tr}S(W \rightarrow NMF + W)_{(A)}$
